# Analysis of Risk Factors for Cholelithiasis: A Single-Center Retrospective Study

**DOI:** 10.7759/cureus.46155

**Published:** 2023-09-28

**Authors:** Anusha Baddam, Ogbonnaya Akuma, Rohan Raj, Chinaza M Akuma, Sana W Augustine, Ihab Sheikh Hanafi, Gauravdeep Singh, Ahmer Zain, Nasihudeen Azizz, Manjeet Singh, Kainat Makheja, FNU Rahul, Aadil Khan

**Affiliations:** 1 Medicine and Surgery, Malla Reddy Medical College for Women, Hyderabad, IND; 2 Internal Medicine, Ebonyi State University, Abakaliki, NGA; 3 Internal Medicine, Nalanda Medical College and Hospital, Patna, IND; 4 Medicine, College of Health Professions, Chamberlain University, Chicago, USA; 5 Internal Medicine, Liaquat University of Medical and Health Sciences, Hyderabad, PAK; 6 Internal Medicine, Spartan Health Sciences University, Vieux Fort, LCA; 7 Medicine, Sigma Diagnostics, Khanna, IND; 8 General Medicine, Kempegowda Institute of Medical Sciences, Bangalore, IND; 9 Internal Medicine, Kempegowda Institute of Medical Sciences, Bangalore, IND; 10 Internal Medicine, Liaquat National Hospital and Medical College, Karachi, PAK; 11 Internal Medicine, Jinnah Sindh Medical University, Karachi, PAK; 12 Trauma Surgery, OSF St. Francis Medical Centre, Peoria, USA; 13 Cardiology, University of Illinois at Chicago, Chicago, USA; 14 Internal Medicine, Lala Lajpat Rai (LLR) Hospital, Kanpur, IND

**Keywords:** risk factors, gall bladder diseases, gallstones, obesity and diabetes, cholelithiasis

## Abstract

Objectives

Cholelithiasis poses a considerable medical burden worldwide. While its pathogenesis is multifactorial, identifying the key risk factors is essential for understanding the disease and improving patient care. This study aims to investigate the potential associations between demographic, clinical, and laboratory variables and the development of cholelithiasis.

Methods

This single-center retrospective study was conducted at Malla Reddy Institute of Medical Sciences, Hyderabad, India, over one month. A total of 200 patients diagnosed with cholelithiasis were included. Data were extracted from electronic health records and the patients using a questionnaire, including demographic information (age, gender), clinical data including body mass index (BMI), and comorbidities. Statistical analyses were conducted to determine the associations between risk factors and cholelithiasis.

Results

The frequency of cholelithiasis is found to be higher in the female gender and patients with obesity, sedentary lifestyle and hypertension as compared to male patients, and the risk of cholelithiasis also increases with age. Females demonstrated a higher prevalence of cholelithiasis, with an odds ratio (OR) and confidence interval (CI) of 1.4, 95% CI [1.1, 1.7], p < 0.05). Obese individuals (BMI ≥ 30) had 2.2 times higher odds of cholelithiasis compared to those with normal BMI (< 24.9) (OR = 2.2, 95% CI [1.7, 2.9], p < 0.001). The presence of diabetes significantly increased the odds of cholelithiasis by 1.6 times (OR = 1.6, 95% CI [1.2, 2.1], p < 0.01). Overweight individuals (BMI: 25-29.9) were associated with 1.4 times higher odds of cholelithiasis (OR = 1.4, 95% CI [1.1, 1.9], p < 0.05).

Conclusion

Our study identified age, gender, BMI, diabetes, and obesity as significant risk factors for cholelithiasis. These findings underscore the importance of targeted interventions and lifestyle modifications to mitigate cholelithiasis risk and improve patient outcomes. Further research, including prospective multicentric studies, must validate these findings and explore potential underlying mechanisms.

## Introduction

Cholelithiasis, the formation of gallstones within the gallbladder or bile ducts, remains a prevalent and clinically significant condition affecting millions worldwide. It is a substantial burden on healthcare systems due to its associated complications, including biliary colic, cholecystitis, and even potentially life-threatening conditions like choledocholithiasis. The etiology of cholelithiasis is multifactorial, involving intricate interactions between genetic, environmental, and lifestyle factors.

Cholelithiasis is the condensation of cholesterol or bile pigments leading to the formation of solid crystal deposits in the gallbladder. The gallbladder stores bile acids and bile salts until they are needed to help digest fatty foods. These depositions can obstruct the bile outflow, resulting in biliary colic, eructation, food intolerance, and severe right upper abdominal pain radiating towards the right shoulder. In the US, between the ages of 20 and 74, 6.3 million males and 14.2 million women have been reported to have gallbladder disease [1]. The incidence of cholelithiasis in non-Western countries is more than 10% in Asia and less than 5% in Africa [2]. Cholelithiasis is predominantly reported in females who are above 40 and obese [3]. With the revolution in chemotherapeutics, the graph of non-communicable diseases is rising. Therefore, it seems essential to identify the preventable factors that enhance the disease incidence. It is associated with multiple risk factors, which can be further divided into modifiable and non-modifiable factors. Modifiable factors include sedentary lifestyle, drugs, rapid weight loss and obesity. In contrast, non-modifiable factors include age, gender, ethnic group and genetic predisposition [4,5]. Increasing age, rural habitat, and diastolic hypertension in China and Taiwan are shown to be associated with gallstone formation.

While considerable progress has been made in understanding the pathophysiology of cholelithiasis, identifying specific risk factors contributing to its development remains an essential research focus. Recognizing these risk factors is crucial for preventative efforts and optimizing patient management strategies. The potential contributors are age, gender, body mass index (BMI), and comorbid conditions such as diabetes and obesity [4]. Understanding the significance of these factors can pave the way for targeted interventions and risk reduction strategies, ultimately improving patient outcomes and alleviating the burden on healthcare systems.

## Materials and methods

Study design and data collection

This study was designed as a monocentric retrospective analysis to identify and analyze the risk factors associated with cholelithiasis. The study was conducted at Malla Reddy Institute of Medical Sciences, India, over a period of one month. A total of 200 patients diagnosed with cholelithiasis in the last five years were included in the study. The patient's medical records were retrieved from the electronic health record system of the medical center from the last five years (2018-2022). The data collection process involved the systematic extraction of relevant information from electronic health records and demographic data such as age, gender, BMI and ethnic background of the patients, and detailed clinical history related to the diagnosis and management of cholelithiasis, including the presence of symptoms, previous medical interventions, and family history of gallstones, comorbidities such hypertension and diabetes mellitus and imaging reports, such as ultrasound, computed tomography (CT), or magnetic resonance imaging (MRI) scans, confirming the presence and characteristics of gallstones.

Inclusion and exclusion criteria

Inclusion criteria involve patients with a confirmed diagnosis of cholelithiasis based on medical records, including imaging reports, indicating the presence of gallstones. Patients aged>15 years at the time of cholelithiasis diagnosis were included, with comprehensive and accessible medical records containing demographic information, clinical history, laboratory results, and relevant imaging findings related to cholelithiasis diagnosis within the predefined period and the patients not undergoing active treatment or surgical intervention for cholelithiasis at the time of data collection were included in our study. Exclusion criteria involve secondary gallstones, age (<15 years), incomplete medical records, treatment influence, and unconfirmed diagnosis. Among variables in our study, the World Health Organization (WHO) criteria for obesity is defined as healthy weight (BMI: 18.5-24.9), overweight (BMI: 25-29.9), and obesity (BMI ≥ 30) [6]. The WHO criterion for hypertension is individuals with a blood pressure of 140/90 mmHg or higher [7].

Data analysis

Statistical analysis was performed using appropriate software (e.g., SPSS [IBM Corp., Armonk, NY, USA], R [R Foundation for Statistical Computing, Vienna, Austria]). Descriptive statistics were calculated for demographic and clinical variables. Data was entered into SPSS version 21.0. The mean, standard deviation (SD), frequencies, and percentages were calculated for variables such as age, gender, mode of admission, and risk factors. We cross-tabled and compared the frequencies of all the variables in both male and female patients. We also calculated the mean and standard deviation of all the variables. The association between age, gender, mode of admission, and their frequencies were represented by a bar chart. Logistic regression models were employed to estimate the odds ratios (ORs) and 95% confidence intervals (CIs) for the identified risk factors while adjusting for potential confounders. Our study followed ethical guidelines and was conducted in compliance with relevant institutional regulations and patient confidentiality standards. All patient data were anonymized and protected to ensure privacy and compliance with ethical standards.

## Results

**Figure 1:**
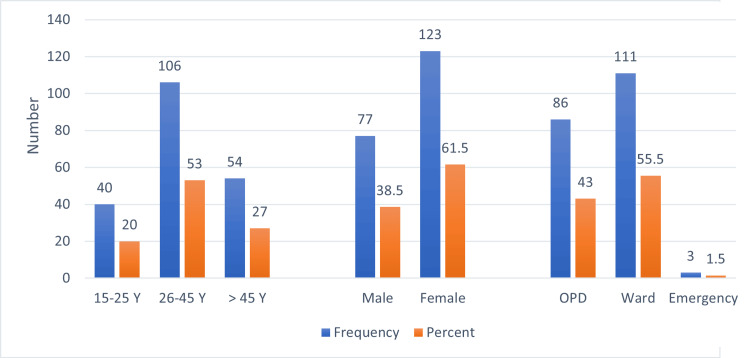
Sociodemographic of study participants. Y: year, OPD: outpatient department

A total of 200 patients were included in our study. The tabulated results of the frequency of socio-demographic factors are shown in Table 1. A total of 53% (n=106) of patients affected with cholelithiasis ranged between 26-45 years of age, and 20% (n=40) of patients ranged from 15-25 years of age. Furthermore, 27% (n=54) of patients were 46 years or older. It is also evident that 61.5% (n=123) of patients were females. Regarding the mode of admission, 55.5% (n=111) of patients were admitted to inpatient wards, and 43% (n=86) of patients were diagnosed in the outpatient department where they were presented with biliary colic. A total of 5% (n=3) of patients presented in the emergency department with acute cholecystitis superimposed on chronic cholelithiasis. The mean age of individuals included in our study was 37.5 (SD: 4.1) years, and the mean age of men and women in our study was 33.2 (SD: 4.3) and 39.5 (SD: 3.2) years. The graphical representation of frequencies of age, sex, and mode of admission of patients is shown in Figure 1.

**Table 1:** Frequency of sociodemographic factors. Data has been presented as N (number) and % (percentage); a p-value of <0.05 is considered statistically significant.

Variables	N	Percent (%)
Age of patient (years)		
15-25	40	20.0
26-45	106	53.0
> 45	54	27.0
Sex of patient		
Male	77	38.5
Female	123	61.5
Admission mode		
Outpatient department	86	43.0
Ward	111	55.5
Emergency	3	1.5

**Figure 2:**
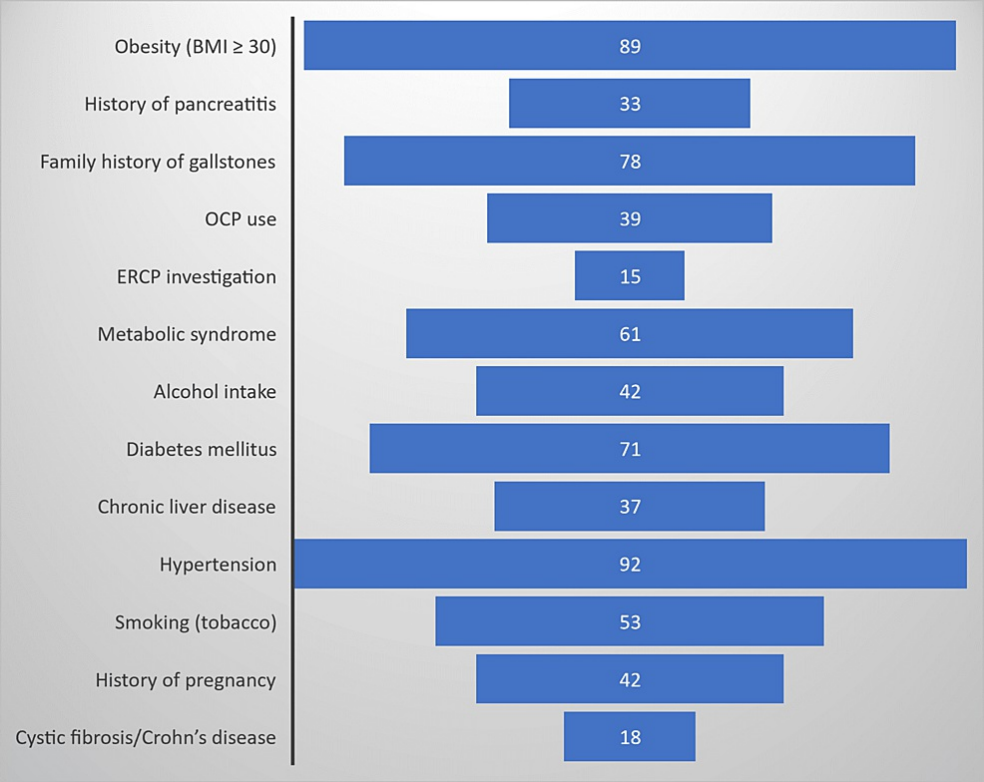
Percentage of risk factors responsible for cholelithiasis. BMI: body mass index, ERCP: endoscopic retrograde cholangiopancreatography, OCP: oral contraceptive pill.

We have tabulated the frequency of risk factors leading to cholelithiasis in Table 2 and Figure 2, which revealed that obesity (BMI≥30) was observed in 89 (44.5%) of the patients, 62 (31%) of the patients were overweight, 71 (35.5%) patients had diabetes, 92 (46.5%) patients had hypertension, and 53 (26.8%) patients had a history of smoking.

**Table 2:** Frequency of risk factors responsible for cholelithiasis. BMI: body mass index, ERCP: endoscopic retrograde cholangiopancreatography, OCP: oral contraceptive pill. Data has been presented as N (number) and % (percentage); a p-value of <0.05 is considered statistically significant.

Risk factor	N	Percentage (%)
Obesity (BMI ≥ 30)	89	44.5%
History of pancreatitis	33	16.5%
Family history of gallstones	78	39.4%
OCP use	39	19.7%
ERCP investigation	15	7.5%
Metabolic syndrome	61	30.8%
Alcohol intake	42	21.2%
Diabetes mellitus	71	35.9%
Chronic liver disease	37	18.5%
Hypertension	92	46.5%
Smoking (tobacco)	53	26.8%
History of pregnancy	42	21.2%
Cystic fibrosis/Crohn’s disease	18	9%

**Figure 3:**
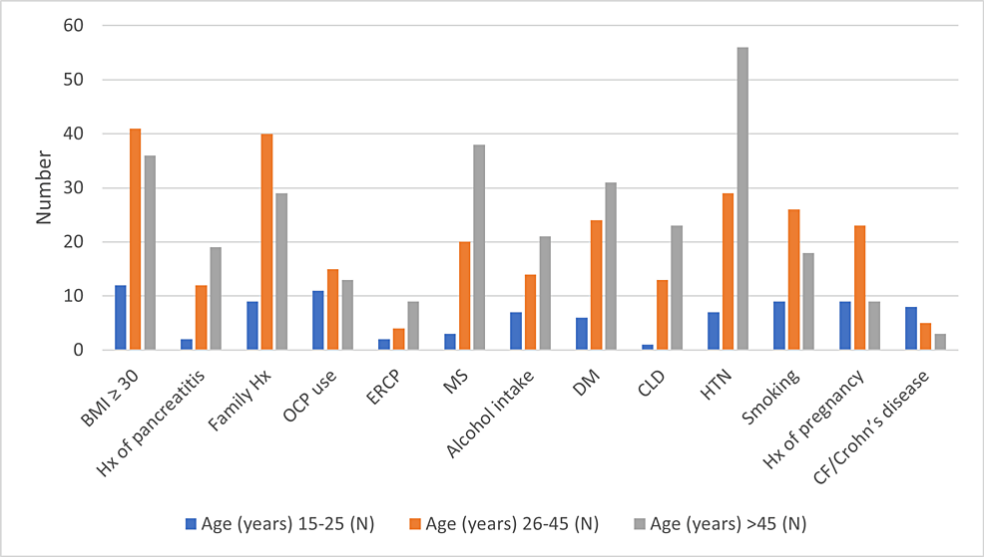
Cross-tabulation of risk factors of cholelithiasis for age BMI: body mass index, ERCP: endoscopic retrograde cholangiopancreatography, OCP: oral contraceptive pill, DM: diabetes mellitus, CF: cystic fibrosis, MS: metabolic syndrome, CLD: chronic liver disease, HTN: hypertension.

Cross-tabulation of risk factors in correlation with age is depicted in Table 3, which shows that the maximum number of patients presenting with gallstones was 25-45 years (Figure 3).

**Table 3:** Cross-tabulation of risk factors of cholelithiasis for age. BMI: body mass index, ERCP: endoscopic retrograde cholangiopancreatography, OCP: oral contraceptive pill, n: number. Data has been presented as N (number); a p-value of <0.05 is considered statistically significant.

Risk factor	Age (years)		
	15-25 (N)	26-45 (N)	>45 (N)
Obesity (BMI ≥ 30)	12	41	36
History of pancreatitis	02	12	19
Family history of gallstones	09	40	29
OCP use	11	15	13
ERCP investigation	2	4	9
Metabolic syndrome	3	20	38
Alcohol intake	7	14	21
Diabetes mellitus	6	24	31
Chronic liver disease	1	13	23
Hypertension	7	29	56
Smoking (tobacco)	9	26	18
History of pregnancy	9	23	9
Cystic fibrosis/Crohn’s disease	8	5	3

**Figure 4:**
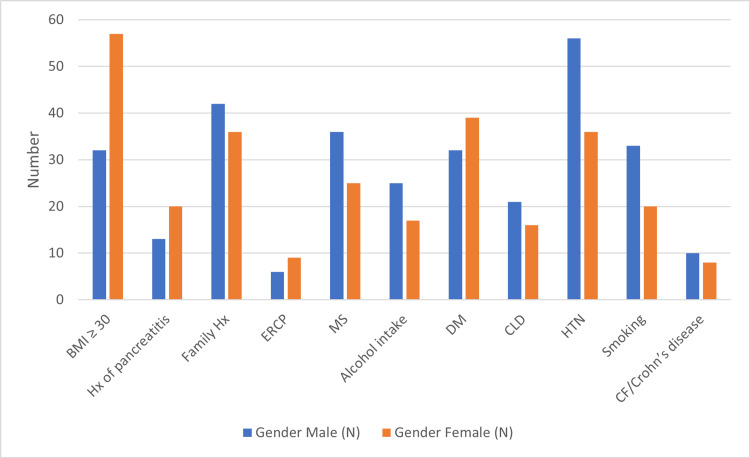
Cross-tabulation of risk factors of cholelithiasis for gender. BMI: body mass index, ERCP: endoscopic retrograde cholangiopancreatography, OCP: oral contraceptive pill, DM: diabetes mellitus, CF: cystic fibrosis, MS: metabolic syndrome, CLD: chronic liver disease, HTN: hypertension.

Cross-tabulation of risk factors with the sex of patients involved in our study sample is shown in Table 4 and Figure 4. If we consider the female gender, plenty of causes were found associated with gallstone formation. Obesity, oral contraceptive (OCP) use, history of pancreatitis, and diabetes mellitus are significant risk factors for cholelithiasis in females. Smoking, alcohol, and metabolic syndrome are the main risk factors responsible for cholelithiasis in males.

**Table 4:** Cross-tabulation of risk factors of cholelithiasis for gender. BMI: body mass index, ERCP: endoscopic retrograde cholangiopancreatography. Data has been presented as N (number); a p-value of <0.05 is considered statistically significant.

Risk factor	Gender	
	Male (N)	Female (N)
Obesity (BMI ≥ 30)	32	57
History of pancreatitis	13	20
Family history of gallstones	42	36
ERCP investigation	6	9
Metabolic syndrome	36	25
Alcohol intake	25	17
Diabetes mellitus	32	39
Chronic liver disease	21	16
Hypertension	56	36
Smoking (tobacco)	33	20
Cystic fibrosis/Crohn’s disease	10	8

A univariate analysis identified potential risk factors for cholelithiasis, which include obesity, hypertension, chronic disease, and family history of gallstone disease (Table 5).

**Table 5:** Univariate logistic analysis of risk factors associated with cholelithiasis. CLD: chronic liver disease, HTN: hypertension, BMI: body mass index, CI: confidence interval, OR: odds ratio, N: number

Variables	Present (N)	Absent (N)	OR	CI	p-value
Obesity (BMI ≥ 30)	89	111	2.2	1.7-2.9	< 0.005
Family history of gallstones	78	122	1.8	1.3.2.1	< 0.05
Metabolic syndrome	61	139	1.3	1.1-1.7	< 0.05
Alcohol intake	42	158	1.1	0.89-1.4	< 0.14
Diabetes mellitus	71	129	1.7	1.2-1.9	< 0.005
CLD	37	163	1.4	1.1-1.7	< 0.005
HTN	92	108	1.6	1.2-2.0	< 0.05
Smoking (tobacco)	53	147	1.4	1.1-1.6	< 0.05

Multivariate analysis of potential risk factors of cholelithiasis was performed using logistic regression, which revealed that the frequency of cholelithiasis is found to be higher in the female gender and patients with obesity, sedentary lifestyle and hypertension as compared to male patients and risk of cholelithiasis also increases with age. Females demonstrated a higher prevalence of cholelithiasis, with an OR of 1.4 (OR = 1.8, 95% CI [1.1, 1.7], p < 0.05). Obese individuals (BMI ≥ 30) had 2.2 times higher odds of cholelithiasis compared to those with normal BMI (< 25) (OR = 2.2, 95% CI [1.7, 2.9], p < 0.001). The presence of diabetes significantly increased the odds of cholelithiasis by 1.6 times (OR = 1.6, 95% CI [1.2, 2.1], p < 0.01). Individuals with overweight (BMI: 25-29.9) were associated with 1.4 times higher odds of cholelithiasis (OR = 1.4, 95% CI [1.1, 1.9], p < 0.05) as compared to those with normal BMI (Table 6). 

**Table 6:** Multivariate regression analysis of potential risk factors of cholelithiasis. CI: confidence interval, OR: odds ratio, DM: diabetes mellitus, BMI: body mass index, Hx: history.

Variables	Positive (%)	Adjusted OR	95% CI	P-value
Sex		1.4	1.1-1.7	< 0.05
Male	38.5			
Female	61.5			
Family Hx of gallbladder stones		1.1	0.81-1.21	< 0.05
Yes	39			
No	61			
Obesity (BMI)		2.2	1.7-2.9	< 0.001
<25	55.5			
≥30	44.5			
DM		1.6	1.2-2.1	< 0.01
Yes	35.9			
No	64.1			
Hypertension		1.2	0.87-1.41	< 0.05
Yes	46.5			
No	53.5			

## Discussion

Cholelithiasis remains a complex and clinically significant condition, and understanding its risk factors is pivotal for effective preventive and management strategies. The results of this study underscore the importance of age, gender, BMI, and comorbidities as significant risk factors for the development of cholelithiasis. The observed significant association between age and cholelithiasis aligns with the cumulative nature of gallstone formation [5]. This finding is consistent with previous research highlighting the increasing incidence of cholelithiasis with advancing age [8]. As age increases, factors such as changes in gallbladder motility, alterations in bile composition, and decreased bile acid synthesis could contribute to stone formation. This relationship underscores the importance of age as a crucial risk factor that should be considered in clinical assessments and preventive efforts [9].

The higher prevalence of cholelithiasis among females, as observed in this study, has been widely documented in existing literature. The potential hormonal influence, particularly estrogen's impact on cholesterol metabolism and gallbladder motility, is thought to play a role in this gender disparity [10]. The risk of cholelithiasis in women is high as compared to men because of increased influence of female sex hormones and increased risk in fertile women who have experienced multiple pregnancies as compared to non-pregnant because of changes in bile composition and gallbladder stasis [11-13]. This evidence is supported by our study, which justified the high prevalence of cholelithiasis in females. This is similar to the use of hormone replacement therapy and oral contraceptives, which also leads to high gallstone formation [14].

Alcohol consumption and increased serum high-density lipoprotein (HDL) levels are inversely related to cholelithiasis [2]. Patients with a history of pancreatitis have eleven times more risk of developing gallstones. High altitude is also related to gallstone formation because of slow intestinal transit that causes constipation and further bilirubin absorption and concentration in the gallbladder [3]. Hemolytic diseases like sickle cell anemia in children and chronic renal failure in adults increase the risk of cholelithiasis [4,10]. In secondary hyperparathyroidism, an increase in serum calcium phosphate and its excretion into bile leads to crystallization. If there is a family history of cholelithiasis, then the risk increases five times. The risk further increases four to 10 times after 40 years of age [5].

The strong association between obesity and cholelithiasis reaffirms the role of excess adiposity in gallstone formation. Obesity triggers various metabolic alterations that increase cholesterol saturation in bile, promoting nucleation and stone growth [15]. Additionally, obesity-related alterations in gut hormones and adipokines may impact gallbladder motility and contribute to gallstone development. The notable odds ratio for obesity emphasizes the significance of weight management as a preventive measure for gallstone formation [16]. Rapid weight loss that exceeds 1.5kg/body weight following bariatric surgery increases the risk of gallstone formation postoperatively. Increased dietary intake of cholesterol, legumes, fatty acids, and carbohydrates increases the risk of cholelithiasis [5]. An increase in BMI is also associated with a higher risk of gallstone formation. A Mendelian randomized study showed a 7% higher risk of gallstone formation with each kg weight loss in persons with measured BMI [12]. This evidence is supported by the results of our study, which reports that as the BMI increases, the likelihood of cholelithiasis also increases.

Chronic diseases like cirrhosis, cystic fibrosis, type 1 sickle, and gauche disease cell anemia are also related to cholelithiasis. In patients with extensive ileal Crohn’s disease, the risk increases by two to three times [5,8,10,17]. Our study highlights the presence of cystic fibrosis and Crohn's disease as justifiable risk factors for cholelithiasis. The associations between diabetes and obesity with cholelithiasis are noteworthy. Diabetes is recognized for its impact on gallbladder motility and bile composition, potentially predisposing individuals to stone formation [18]. The combination of diabetes and obesity could exacerbate these effects, amplifying the risk. These findings emphasize the importance of addressing metabolic health in individuals with diabetes to mitigate cholelithiasis risk. The observed link between obesity and diabetes in this study further underscores the complex interplay of these risk factors [19-21].

The identification of these risk factors holds significant clinical implications. Firstly, clinicians should be vigilant in assessing age, gender, BMI, and comorbidities when evaluating individuals for cholelithiasis risk [22,23]. This understanding can aid in early detection, risk stratification, and tailored patient counselling. For instance, obese individuals with comorbid diabetes might benefit from targeted interventions focusing on weight reduction and metabolic control [24]. Similarly, females may require specific hormonal evaluations to assess potential influences on gallstone development. The findings of this study lend support to the development of preventive strategies. Public health initiatives can emphasize weight management programs, especially for individuals with obesity, to mitigate the risk of gallstone formation. Similarly, promoting lifestyle modifications to address comorbidities like diabetes could impact overall health and gallstone risk reduction [25].

Our study has certain limitations that should be acknowledged. The retrospective design inherently risks selection bias and incomplete data capture. While efforts were made to include a diverse patient cohort, the monocentric nature of the study could limit the generalizability of the findings to broader populations. Lifestyle factors, such as dietary habits and physical activity recognized contributors to gallstone formation, were not extensively explored due to data limitations.

## Conclusions

Our study contributes to the understanding of risk factors associated with cholelithiasis. The identified risk factors, including age, gender, BMI, and comorbidities like diabetes and obesity, shed light on the complex interplay between genetic, hormonal, metabolic, and lifestyle factors in gallstone formation. These findings hold implications for clinical practice, public health initiatives, and future research directions aimed at alleviating the burden of cholelithiasis on individuals and healthcare systems. By addressing these risk factors, healthcare professionals can work towards proactive strategies that enhance patient outcomes and quality of life. The findings of this study have the potential to enhance our understanding of cholelithiasis etiology, thereby guiding clinical practice and informing public health initiatives.
